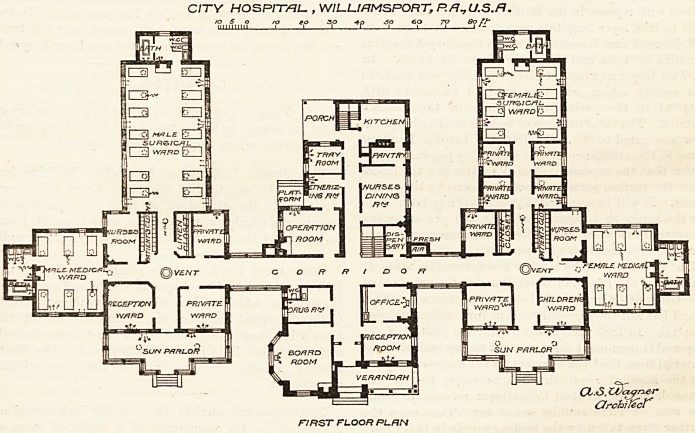# Hospital Construction

**Published:** 1899-09-23

**Authors:** 


### The Institutional Workshop.

### HOSPITAL CONSTRUCTION.

### THE CITY HOSPITAL FOR WILLIAMSPORT, PA., U.S.A.

This hospital was organised by a committee appointed by the County Medical Society in 1873. The charter required twelve managers, seven of whom had to be members of the society living in the city. To this clause in the charter the hospital owes its freedom from interference by irregular practitioners. The present medical staff consists of four physicians and four surgeons. There is also a dispensary staff of six younger physicians, who serve daily for two months at a time, and from amongst these younger physicians the vacancies in the higher staff are filled—merit, not length of service being the basis of election. There are two physicians resident at the hospital. The present building was opened in 1890. It is built on high ground just outside of Williamsport, and the site has the advantage of being surrounded with fine old oak trees. The main elevation faces due south. The building consists of three blocks, joined to each other by corridors. The central block contains the administrative department, and comprises, on the ground floor, board-room, operating-room, nurses' dining-room, reception-room, kitchen, &c. The first floor of the central block contains the lying-in-rooms, bath-rooms, and private wards, while the second floor is given up to the nurses for their quarters. East of the central block is the block for men. It is divided into two wards medical and surgical. The latter contains fourteen beds, and the former six. Both are of good design, but as the medical ward is so much smaller than the surgical one, and as it is dwarfed by a three-storey building, so near, we think it would have been an improvement to have separated it from the main block by a corridor ten or twelve feet long. The closet blocks project from the ends of the wards, but they are not separated by ventilating corridors from the wards as they ought to have been. This east wing also contains two private wards, a reception-room, and a nurses' room; and the south aspect of the block ends in an enclosed verandah or "sun parlor," as the architect calls it. This must be a feature of the block at once striking and useful. The women's block is almost the same as the men's; the only difference is that the surgical dormitory contains only eight beds, the rest of the space being taken up by four private wards, or single-bedded rooms. We do not notice any laundry. Generally, it may be said that the hospital is distinctly above the level of its fellows of similar size (it seems to contain about 50 beds), and, with the alterations we have referred to, it would have been really good. The building materials used are press bricks and stone dressings, such being very suitable to the "domestic" style of architecture employed. The hospital is warmed by hot air, but we are glad to note that open fires are also used. It is proposed to build another block, and new operating-rooms are to be added to the central block, and one of the present rooms is to be fitted up for operations on the lungs and the abdomen. A training school for nurses exists in the hospital. The cost of maintenance per bed occupied is about 3s. 9d. a day.

CITY HOSPITAL, WILLIAMSPORT, P.A., U.S.A.

FIRST FLOOR PLAN

## Figures and Tables

**Figure f1:**